# Genome-wide analysis of R2R3-MYB transcription factors in *Boehmeria nivea* (L.) gaudich revealed potential cadmium tolerance and anthocyanin biosynthesis genes

**DOI:** 10.3389/fgene.2023.1080909

**Published:** 2023-02-21

**Authors:** Xinkang Feng, Aminu Shehu Abubakar, Kunmei Chen, Chunming Yu, Aiguo Zhu, Jikang Chen, Gang Gao, Xiaofei Wang, Pan Mou, Ping Chen

**Affiliations:** ^1^ Institute of Bast Fiber Crops, Chinese Academy of Agricultural Sciences, Changsha, China; ^2^ Department of Agronomy, Bayero University, Kano, Nigeria

**Keywords:** ramie, MYB TFs, expression profiles, cadmium stress, anthocyanin biosynthesis, protein interaction

## Abstract

Gene family, especially MYB as one of the largest transcription factor family in plants, the study of its subfunctional characteristics is a key step in the study of plant gene function. The sequencing of ramie genome provides a good opportunity to study the organization and evolutionary characters of the ramie MYB gene at the whole genome level. In this study, a total of 105 BnGR2R3-MYB genes were identified from ramie genome and subsequently grouped into 35 subfamilies according to phylogeny divergence and sequences similarity. Chromosomal localization, gene structure, synteny analysis, gene duplication, promoter analysis, molecular characteristics and subcellular localization were accomplished using several bioinformatics tools. Collinearity analysis showed that the segmental and tandem duplication events is the dominant form of the gene family expansion, and duplications prominent in distal telomeric regions. Highest syntenic relationship was obtained between BnGR2R3-MYB genes and that of *Apocynum venetum* (88). Furthermore, transcriptomic data and phylogenetic analysis revealed that *BnGMYB60*, *BnGMYB79/80* and *BnGMYB70* might inhibit the biosynthesis of anthocyanins, and UPLC-QTOF-MS data further supported the results. qPCR and phylogenetic analysis revealed that the six genes (*BnGMYB9*, *BnGMYB10*, *BnGMYB12*, *BnGMYB28*, *BnGMYB41*, and *BnGMYB78*) were cadmium stress responsive genes. Especially, the expression of *BnGMYB10/12/41* in roots, stems and leaves all increased more than 10-fold after cadmium stress, and in addition they may interact with key genes regulating flavonoid biosynthesis. Thus, a potential link between cadmium stress response and flavonoid synthesis was identified through protein interaction network analysis. The study thus provided significant information into MYB regulatory genes in ramie and may serve as a foundation for genetic enhancement and increased productivity.

## Background

The TFs are generally made up of four functional domains regions that include DNA binding domain, transcription regulation domain, oligomerization site, as well as the nuclear localization signal based on which as well as the number domain residues TFs were classified (Sacks et al., 2018). MYB TFs being unarguably the largest family are distinguished by its highly conserved MYB domain composed of one to four adjacent imperfect tandem repeats at the N-terminus (Riechmann and Ratcliffe, 2000). Each repeat is approximately 50–53 amino acid residues in length, containing three α-helices that together form a helix-turn-helix (HTH) secondary structure that interacts with the major DNA at the specific recognition site C/TAACG/TG during transcription (Dubos et al., 2010). On the contrary, the C-terminal region is highly variable that leads to the wide range of regulatory roles of the MYB gene family (Ogata et al., 1996). According to the number of the adjacent MYB repeats, MYB genes are categorized into four subfamilies: 1R-MYB, R2R3-MYB, R1R2R3-MYB, and 4R-MYB. The R2R3-MYB are generally the most diverse and extensively studied group (Jiang and Rao, 2020). This also led to the subfunctionalization of the large MYB family (Wani et al., 2018). For example, the typical subfamily S6, S5, S4, S7, S44, and S79 participated in the regulation of flavonoid biosynthesis. Representative S1, S2, S11, S17, S20, S22, and S38 subfamilies that contribute to biological stress response, and S9, S15, S18, S25, and S27 subfamilies that are related to growth and development (Gahlaut et al., 2016). In addition, the experimental data of S16, S24, S28, S31, S33, S36, and S37 and other subfamilies are limited and need further research (Wu et al., 2022).

The MYB TF is present in all eukaryotes and was first reported in 1987 in *Zea mays* which was functionally characterized to partake in regulating anthocyanin biosynthesis (Paz-Ares et al., 1987). Nowadays, with the advance of plant genome-wide association and molecular biology methods, an increasing number of studies have shown MYB proteins to play significant role in regulating plant primary and secondary metabolism, plant development, and in the response to various biotic and abiotic stresses (Feller et al., 2011; Baldoni et al., 2015; Ng et al., 2018; Li et al., 2019). For example, *CsTSI* regulates theanine biosynthesis (Zhang et al., 2020), *CaMYB31* regulates capsaicin biosynthesis (Arce-Rodríguez and Ochoa-Alejo, 2017), *DkMyb4* regulated proanthocyanidin biosynthesis in persimmon (Akagi et al., 2009). In addition, many MYBs associated with anthocyanin biosynthesis have been found in many species in recent years, such as Arabidopsis (*AtMYB75, AtMYB90, AtMYB113* and *AtMYB114*) (Bac-Molenaar et al., 2015; Jian et al., 2019; Muñoz-Gómez et al., 2021), citrus (*CsRuby1* and *CsMYB3*) (Huang et al., 2020) and tomato (*SlMYB12*) (Ballester et al., 2010). They interact with anthocyanin synthesis pathway genes through MBW complex to form anthocyanin synthesis regulatory network (Lloyd et al., 2017). The resistance mechanism mediated by MYB has also been reported. For example, *GbMYB5* confers drought tolerance in cotton and transgenic tobacco plants (Chen et al., 2015a), another rice MYB (*OsMYB3R-2*) exhibited enhanced cold tolerance by alteration in cell cycle and ectopic expression of stress genes (Ma et al., 2009). To resist soil cadmium contamination, plants have evolved fine-tuned mechanisms to protect cells from Cd toxicity by partitioning Cd into vacuoles or trapping free Cd^2+^ in the cytosol (Clemens and Ma, 2016). Studies from several independent laboratories have demonstrated the roles of HIPPs in heavy metal transport, accumulation, and detoxification (Chu et al., 2005; Tehseen et al., 2010; Zhao et al., 2013). In addition, *AtMYB59* is involved in plant growth and cadmium stress response in *Arabidopsis Thaliana* by acting as negative regulator of Ca signaling and homeostasis (Fasani et al., 2019). *AtMYB4* regulates cadmium tolerance by enhancing protection against oxidative damage and increasing expression of *phytochelatin synthase 1* (*PCS1*) and *metallothionein 1C* (*MT1C*) (Agarwal et al., 2020). Other studies have shown that ABA is a feedback mechanism that controls Cd uptake and accumulation in plant cells (Hsu and Kao, 2003). Induced by cadmium stress, ABI5 is upregulated and interacts with *AtMYB49*, preventing its binding to downstream gene promoters, thereby reducing Cd accumulation. On the other hand, *AtMYB49* positively regulates the expression of *bHLH38* and *bHLH101*, resulting in the activation of a metal transporter involved in Cd absorption (Zhang et al., 2019). Interestingly, these mechanisms do not exist independently. A recent study from the perspective of root development in response to abiotic stress revealed that *MYB36* balances root lignification and suberization by mediating the *MYB74-MYB36-MYB92-MYB93* subnetwork (Xu et al., 2022). The above studies indicate that the functions of MYB transcription factors are diversified, gradually forming a complex network mechanism.

Ramie is an important fiber crop in the Urticaceaes family which is also widely used as feed and other industrial raw materials (Kiprioti et al., 2015; Mu et al., 2020; Wang et al., 2021). In addition, its perennial nature makes it extremely resistant, and its high cadmium tolerance makes it a soil remediation crop in mining areas in southern China. The diverse functions of R2R3-MYB can connect a variety of regulatory metabolic networks. Therefore, we proposed to study the functional differentiation of MYB family genes in secondary metabolism and abiotic stress. Despite the important role in plant specific processes, very limited reports are present to date on the functional characteristics of R2R3-MYB transcription factor in ramie. Therefore, this study comprehensively investigated and reported gene structure, gene duplication, chromosomal localization, phylogeny, and cis-acting elements of the BnGR2R3-MYB gene. Transcriptome expression profiling coupled with UPLC-QTOF-MS was used to identify BnGMYBs involved in anthocyanin synthesis. Two ramie varieties (HX_1 and ZZ_1) were exposed to cadmium stress and expressions of some selected MYBs explored to understand their function in stress responses in the ramie. Protein interaction network analysis in addition, revealed a potential link between cadmium stress and flavonoid synthesis.

## Results and discussion

### Identification and sequence feature of ramie MYB genes

To identify the MYB family genes in ramie genome, the amino acid sequence of hidden Markov model (HMM) profile of the Pfam MYB domain (PF00249) was used as a query and obtained 245 amino acid sequences that contain MYB or MYB-like repeats. Subsequent processing with FGENESH-M returned 211 sequences. After confirmation of the presence of MYB domains base on the PROSITE, Pfam and NCBI-CDD analyses, we finally established a total of 200 non-redundant MYB proteins which included 89 1R-MYB, 105 R2R3-MYB, 5 R1R2R3-MYB and 1 4R-MYB proteins. According to previous reports, R2R3-MYB family members not only represent more than half of the proportion of the total MYB proteins, but also contain most functional genes, such as specialized metabolism, including the benzenoid, phenylpropanoid, terpenoid, and glucosinolate (GSL) pathways (Dubos et al., 2010). Therefore, we selected only the R2R3-MYB for further study. Based on their order on the chromosome, the R2R3-MYB genes (BnGMYBs) were renamed *BnGMYB1* to *BnGMYB102*. Three of the 105 R2R3-MYBs (*Maker00022368*, *Maker00031449*, and *Maker00022368*) which could not be conclusively mapped to any chromosomes, were renamed *BnGMYB103*—*BnGMYB105* respectively (Supplementary Table S1). This may be due to a large number of gaps, faulty assembly or contamination of organelle DNA and circular DNA *in vitro*. The molecular weight of the protein as obtained using ProtParam analytical tool revealed *BnGMYB52* as the smallest with 135 amino acids (aa), and *BnGMYB1* (593 aa) the largest. The MW of the proteins ranged from 15.6 to 65.0 kDa, and the pI ranged from 4.56 (*BnGMYB23*) to 10.08 (*BnGMYB96*). All the R2R3 but two are localized in the nuclear region, whereas the remaining two were in either chloroplast or cytoplasm (Supplementary Table S1).

**FIGURE 1:**
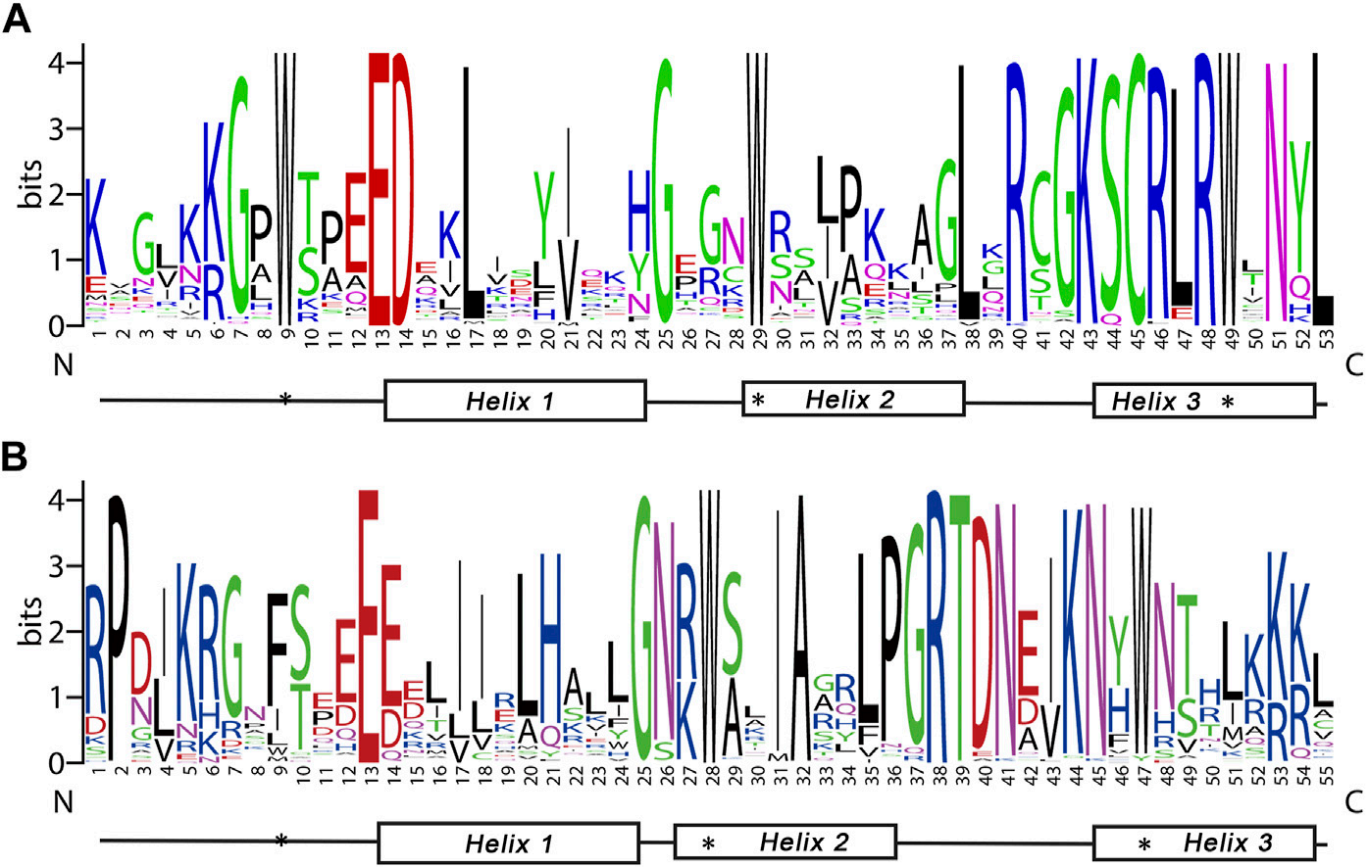
Consensus sequence and the level of conservation of R2R3-MYB domains from ramie. The sequence logos of the R2 **(A)** and R3 **(B)** MYB repeats are based on full-length alignments of all ramie R2R3-MYB domains. The bit score exhibits the information content for each position in the sequence. The position of the three α-helices were marked (Helix 1–3). The conserved tryptophan residues (Trp, W) in the MYB domain are marked with black asterisks.

R2, R3 repeats are significantly conserved sequences within the MYB domain regions. Sequence logos were constructed to investigate the homologous domain features and the frequency of amino acids (Figure 1). There were 108 amino acids conserved residues in the BnGR2R3-MYB. 11 residues in the R2 and 6 in the R3 were completely among all BnGMYBs. These included the most prominent series of evenly distributed and highly conserved triplet tryptophan (Trp., W) residues in each repeat at positions 9, 29 and 49 of the R2 repeat; 28 and 47 of the R3 repeat. These characteristic amino acids are known to play an important role in sequence-specific DNA binding, and are considered landmarks for plant MYB proteins (Jiang et al., 2004). R3 repeats in *BnGMYB66*, *BnGMYB56*, *BnGMYB67* have the last Trp residue replaced with Tyrosine (Tyr., Y) or phenylalanine (Phe., F) which were consistent with those of other species (Li et al., 2016; Liu et al., 2020). Insertion of leucine residue (Leu., L) between second and third helices of the R2 repeat have been reported to represents evolutionary relationship of R2R3-MYB domain (Williams and Grotewold, 1997). In this study, 83 (79.05%) BnGR2R3-MYBs were observed to have Leu-38 inserted between the two helices (Supplementary Table S2).

### Gene structure and motif composition of BnGMYBs

**FIGURE 2:**
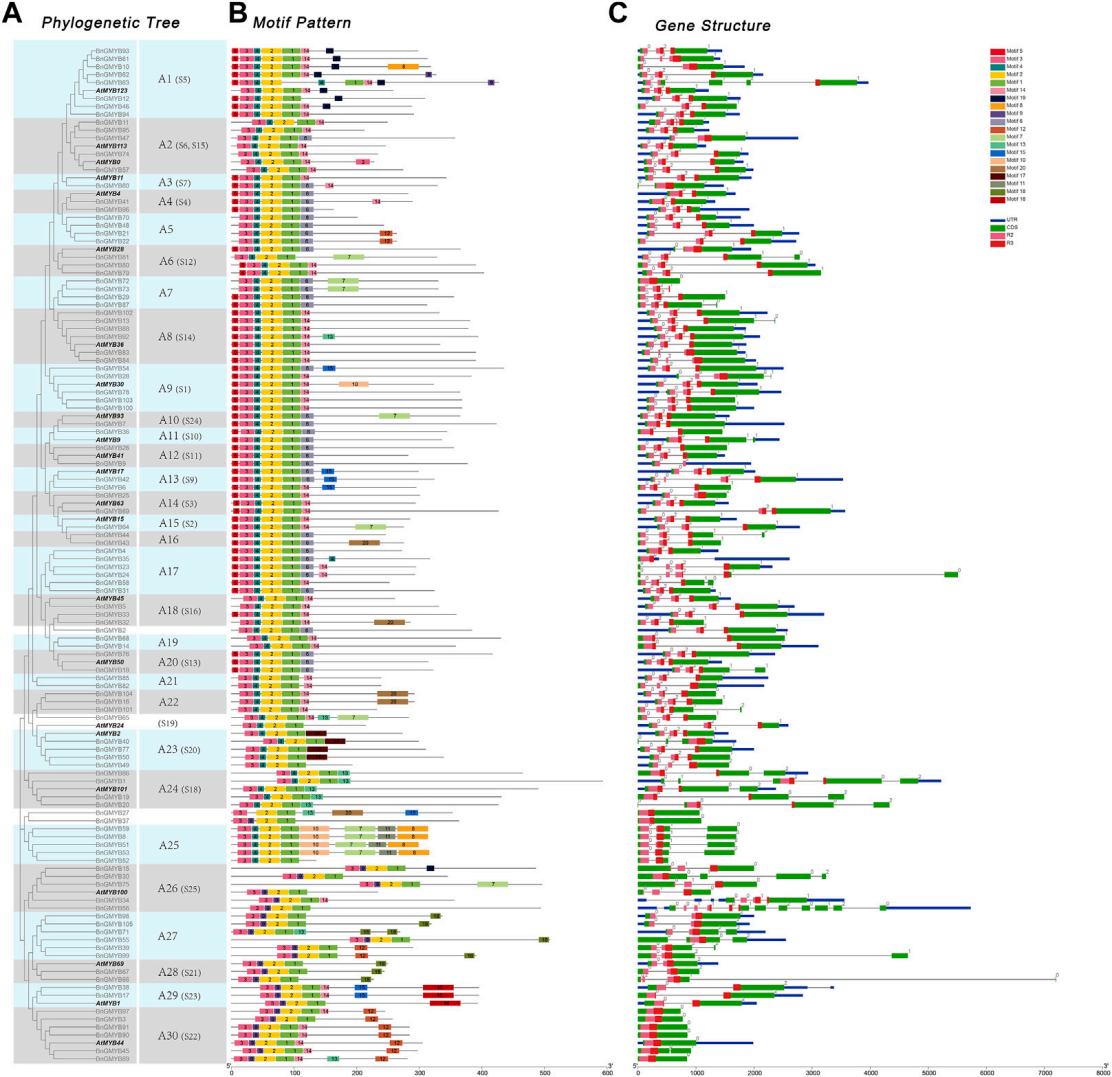
Phylogenetic relationships, gene structure and architecture of conserved protein motifs in BnGR2R3-MYB genes. **(A)** The maximum likelihood phylogenetic includes 105 R2R3-MYB proteins from ramie and 23 representatives from Arabidopsis **(B)** The motif architecture of ramie MYB proteins. 20 different motifs are displayed in different colored boxes. **(C)** Exon-intron structure of ramie MYB genes. Green boxes indicate exons; blue boxes indicate untranslated 5′- and 3′- regions; black lines indicate introns. R2, R3 MYB domain are highlighted by boxes of different shape and color.

We performed a phylogenetic analysis of R2R3-MYB proteins using neighbor-joining (Guo et al., 2019) and maximum likelihood (ML) methods with 1,000 bootstrap replicates. The tree topologies obtained using the two methods were largely similar with a very few variations in the gene classification (Figure 2A and Supplementary Figure S1). Since the ML method can choose the optimal alternative model and optimize the evolutionary tree with certain topological structure and branch length, we adopted it for further characterization. The R2R3-MYB members of ramie were subdivided into 30 subgroups (designated A1-A30 in this study) using Arabidopsis MYB proteins as reference (Dubos et al., 2010). Nine clades only contained ramie R2R3-MYB members, and four genes did not fit into any subgroup.

To investigate the relationship between gene structural function and evolution, we analyzed the structural diversity of the BnGR2R3-MYB family, which showed genes having the same genetic structure clustering together as obtained in the phylogenetic tree (Figure 2A). MYB conservative motifs as obtained using MEME online tool (Figure 2B) revealed the presence of 20 conserved motifs in the ramie MYB proteins (Supplementary Table S3). The gene structure analysis showed most of gene coding sequences disrupted by introns, except for almost all members from A30 subgroup, ungrouped *BnGMYB27/37* and *BnGMYB72* (Figure 2C). The number of introns ranges from zero to eleven, with most genes having three exons and two introns in line with previous reports (Du et al., 2012a). Interestingly, most intron insertions occur in R2, R3 conserved domains indicating the important role of these conserved domains in plants. The highly conserved gene structure and domain influence each other, which provided important evidence for the division of subgroups. Similarly, protein architecture was conserved within a specific subgroup. Phylogenetic tree, gene structure, and motif analysis suggested similar functions for MYB proteins within the same subgroup. In addition to the conserved motif representing R2R3, other similar motifs were shared by the BnGR2R3-MYB members within the same subgroup, for example, motifs 11, 17, and 19 were unique to members in clades A25, A23, and A1 respectively.

### BnGMYB genes chromosome distribution and synteny

**FIGURE 3:**
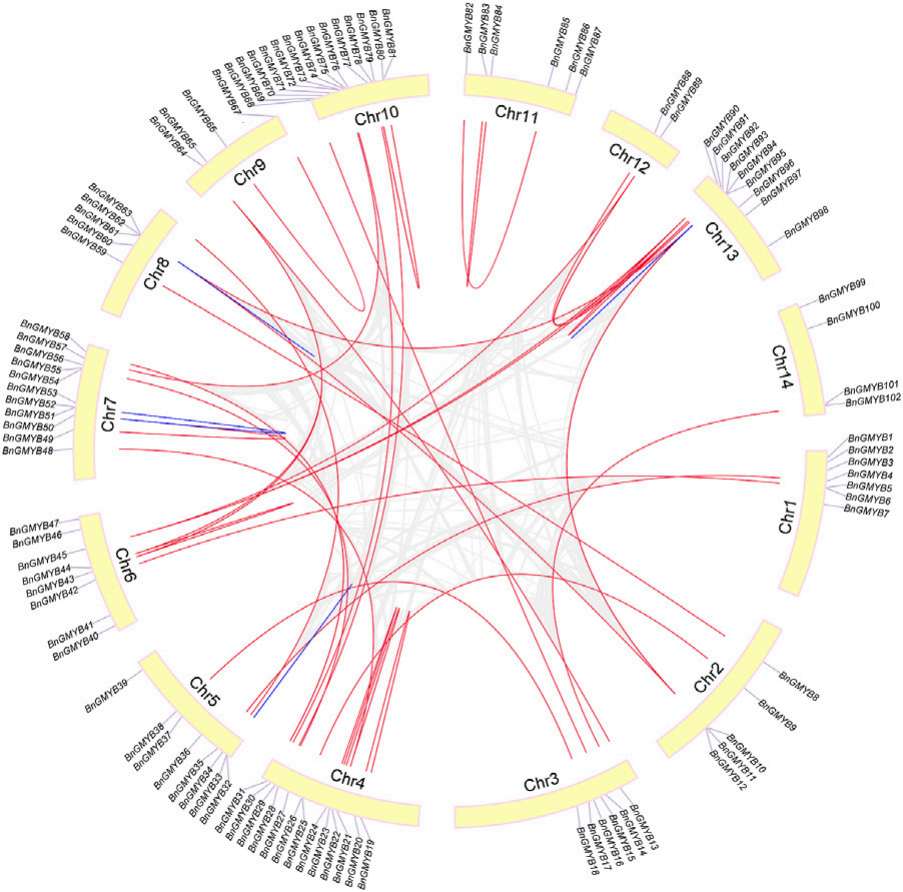
Schematic representations for the chromosomal distribution and inter-chromosomal relationships of ramie R2R3-MYB genes. Gray lines indicate all synteny blocks in the ramie genome, and the red lines indicate duplicated MYB gene pairs. Blue lines indicate tandemly duplicated gene pairs. Gene and chromosome names are displayed on the tip and inside of the chromosome, respectively.

Ramie R2R3-MYB genes were distributed unevenly in the species 14 chromosomes (Figure 3), with Chr10 having the largest number (14) followed by Chr4 (13) and the least number (2) found on Chr12. No significant correlation was obtained between the chromosome length and the number, while some of the genes were concentrated at either top or bottom of the chromosome. The closer the genes are to each end, the more likely they are to crossover (Schilling et al., 2020).

Based on BLASTP and MCScanX methods, we investigated the gene duplication events. Reference to Holub (Holub, 2001) for a description of tandem duplication, among the BnGR2R3-MYB genes, the blue lines in Figure 3 showed four pairs of tandemly duplicated genes (*BnGMYB32/33*, *BnGMYB51/52*, *BnGMYB61/62*, *BnGMYB93/94*) located in Chr5, Chr7, Chr8, and Chr13, respectively, whereas the red lines showed 39 segmental duplication pairs between BnGR2R3-MYB genes (Supplementary Table S4) including two MYB-related genes (*Maker00025472*; *Maker00000772*) and one 3R-MYB gene (*Maker00072694*). Some BnGMYBs located on Chr13, Chr6, and Chr4, such as *BnGMYB91*, *BnGMYB90*, *BnGMYB89*, *BnGMYB21*, *BnGMYB44,* and *BnGMYB45*, were involved in multiple duplication events. *BnGMYB89* (Chr12) was however interrelated with *BnGMYB90/91* (Chr13) and *BnGMYB45* (Chr6). Interestingly, all segmentally duplicated genes fall into A30 subgroups together with *A. thaliana AtMYB44* (Figure 2A) making us speculate that this specific population of R2R3-MYB genes duplication in ramie might be closely related to stress response (Kranz et al., 1998; Shim and Choi, 2013; Nguyen and Cheong, 2018), and might play a vital role in the growth and development of the plant. The segmental and tandem duplication events are major derivers of the gene family expansion (Cannon et al., 2004). The positive pressure criteria were selected based on M. Lynch (Lynch and Conery, 2000): Ka/Ks < 1 stands for purifying selection, Ka/Ks = 1 means neutral selection, while Ka/Ks > 1 signifies positive selection. We found a high sequence divergence value among the four segmental gene pairs. These sequences diverged greatly, and evolution distance was long, owing to the occurrence of a large number of synonymous mutations. The remaining segmental and tandem duplicated BnGMYB gene pairs were Ka/Ks < 1 except *BnGMYB66/67* (Ka/Ks = 1.04). These results suggest that the ramie R2R3-BnGMYB gene family had evolved under the effect of purifying selection (Supplementary Table S4).

**FIGURE 4:**
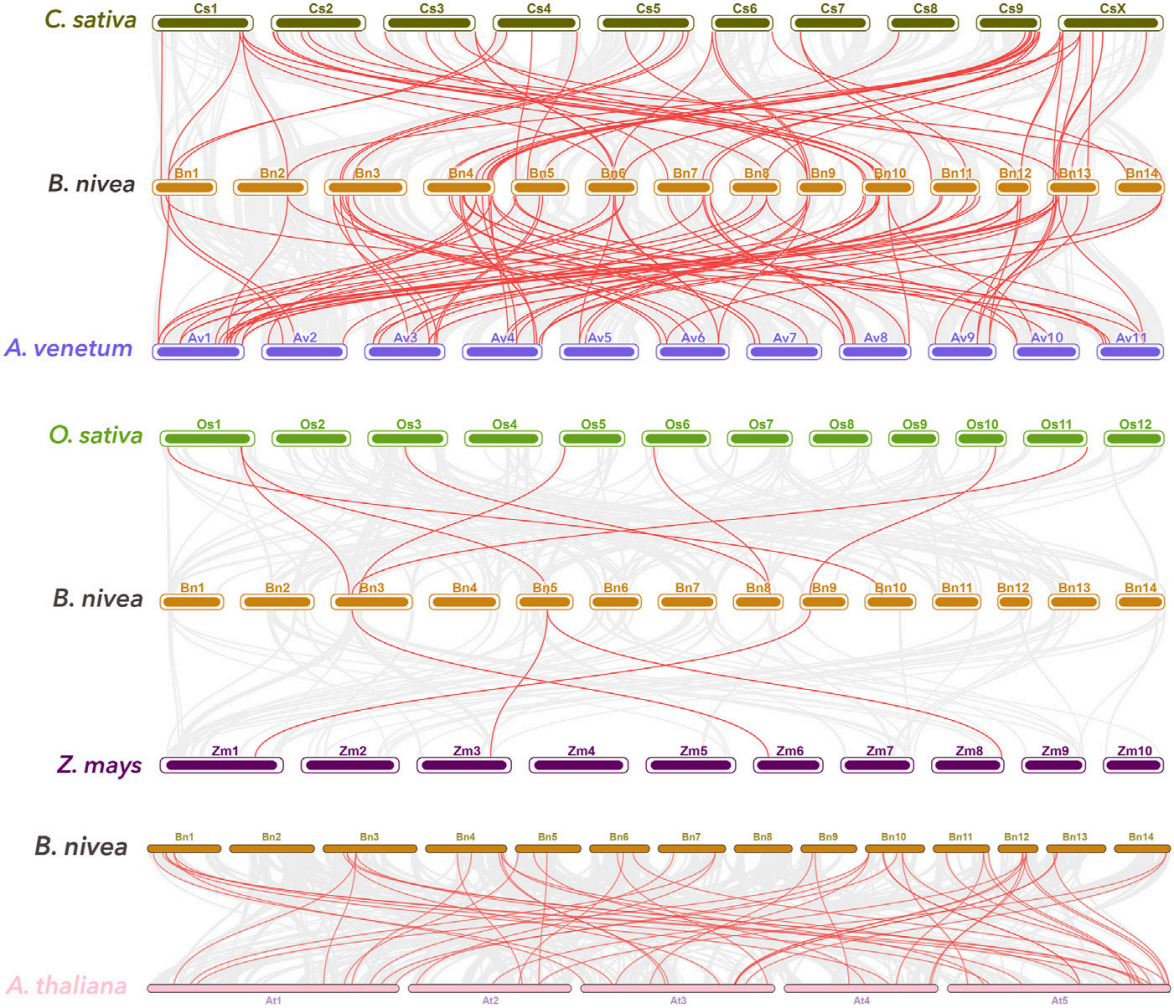
Synteny analyses of R2R3-MYB genes between ramie and five representative plants: *Boehmeria nivea*, *Cannabis sativa*, *A. venetum*, *O. sativa*, *Zea mays* and *A. thaliana*. Gray lines in the background indicate the collinear blocks within ramie and other plant genomes, while the red lines highlight the syntenic R2R3-MYB gene pairs.

Syntenic maps of ramie with five other plants, comprising dicots [*A. thaliana, Cannabis sativa* (*C. sativa* L.)*, A. venetum* (*Apocynum venetum* L.)] and monocots (rice and maize) conducted to further explore potential evolutionary mechanisms of BnGR2R3-MYB genes, revealed 88, 58, 53, 8, and 4 numbers of orthologous gene pairs (Figure 4). Collinearity analysis showed highest collinearity segments of the ramie with *A. venetum and C. sativa* in consistent with the phylogenetic divergence time estimation which supported the placement of ramie within the same clade as *C. sativa* (Luan et al., 2018). Some of the BnGR2R3-MYBs were found to be associated with multiple syntenic gene pairs, particularly *BnGMYB89* and four *A. thaliana* genes, suggestion that these genes might have played an important role in MYB gene family during evolution. Remarkably, the number of collinear gene pair between ramie and *A. venetum*, *C. sativa*, and *A. thaliana* (dicotyledonous) was far greater than between ramie and *Oryza sativa/Z. mays* (monocotyledonous) (Liu et al., 2018; Luan et al., 2018; Huang et al., 2019). 19 BnGR2R3-MYBs identified in dicotyledonous plant were absent in monocotyledonous plant, suggestion that these orthologous pairs (19 BnGR2R3-MYBs) formed after the divergence of dicotyledonous and monocotyledonous plants. Additionally, one collinear pair (*BnGMYB64*) was identified in all the six detected plants, indicating that these orthologous pairs might had already existed before the ancestral divergence (Supplementary Table S5).

### Comparative phylogenetic analysis of BnGMYB and R2R3-MYB family from six different plant species

**FIGURE 5:**
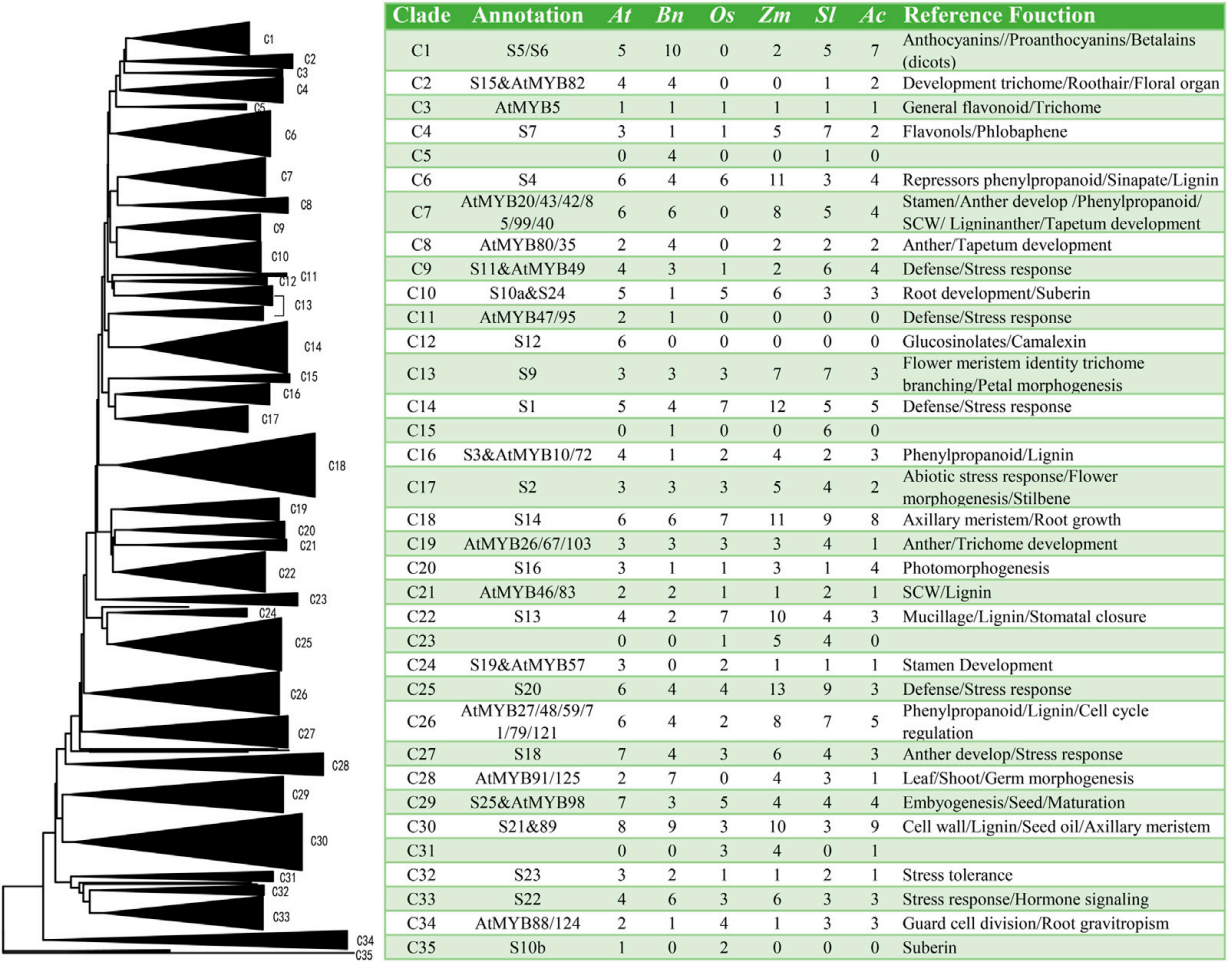
Neighbor-joining (Guo et al., 2019) tree showing relationships among R2R3-MYB proteins from *Boehmeria nivea* (Chen et al., 2015b), *A. thaliana* (*At*), *O. sativa* (*Os*), *Zea mays* (Pierrugues et al., 2001), *S. lycopersicum* (*Sl*) and *A. comosus* (*Ac*). MYB proteins from the six species were designated as C1 to C35. The table on the right indicated the number of the subgroup members and function of clades in each species.

A Neighbor-joining (Guo et al., 2019) phylogenetic tree was constructed using the 105 full-length R2R3-MYB protein sequences of ramie and those from *Arabidopsis* (126), rice (81), maize (156), tomato (121) and pineapple (93) and the result shown in Figure 5 and S2. The phylogenetic analysis generated 35 clades (C1-C35) among six species and the numbers of the MYB members in each species were listed (Figure 5). The fact that this tree was in good agreement with the classification results of *A. thaliana* (Dubos et al., 2010) and other plants (Li et al., 2016; Liu et al., 2017; Liu et al., 2020) demonstrated the reliability of the data. Moreover, the comprehensive phylogenetic analysis of the R2R3-MYB proteins in these six species might provide more evolutionary historical clues. For example, monocotyledon MYB proteins in the same group were often found clustered together, while MYB proteins from dicotyledonous plants were clustered side by side in lateral branches. Meanwhile, several clades were found only in some particular species. For example, none of rice, maize, or pineapple MYB members fall in the C5, C11, and C16. Similarly, C31 had only monocotyledons. This indicated a great evolutionary distance between monocotyledons and dicotyledons, and that these specific MYB genes might have evolved after divergence of the two classes. Apart from these, all other clades contained at least a member from each of the six species, suggesting common ancestry before the species diverged. The varying number of MYBs from the species in a particular clade is an indicative that MYB genes expansion may be more active in some plant species, and this expansion inequality may be related to the well conserved chromosome karyotype (Ming et al., 2015). Furthermore, MYBs in the same clade may have similar function with to the model Arabidopsis, thus provide direction for functional identification of ramie MYB proteins (Kranz et al., 1998; Stracke et al., 2001; Dubos et al., 2010). reference Polyploidy and subsequent genes loss exist in most species is an important driving force of species evolution. Based on the comparative genomic analysis, it could be inferred (Supplementary Figure S3) that ramie has undergone at least one round of genome-wide duplication events. Phylogenetic analysis of MYB gene family showed that the number of BnGMYB members in some clades was consisted with rice and maize. Ramie also undergone the genome-wide duplication event common to rice (Thiel et al., 2009) and maize (Si et al., 2019).

### Analysis of the cis-acting elements of the BnGR2R3-MYB genes and gene ontology annotation

**FIGURE 6:**
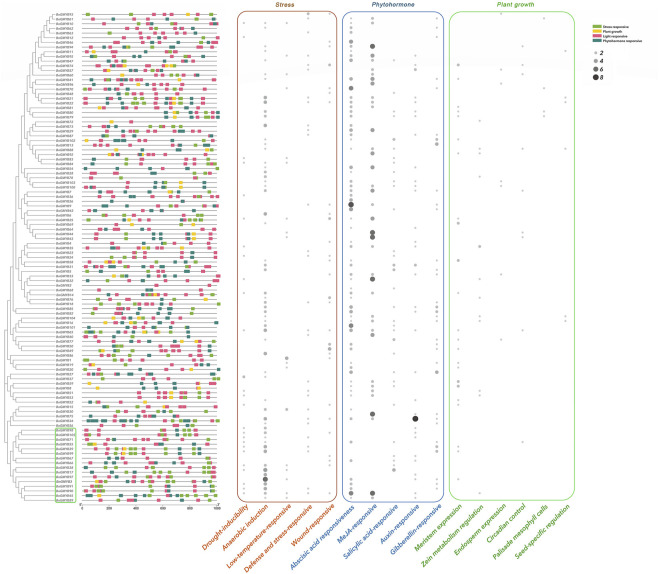
Analysis of cis-elements from promoter region of the BnGR2R3-MYB genes. The figure on the left shows the distribution of four cis-acting elements upstream promoter regions (1000 bp). On the right is the heat map of the quantity of plant growth, stress responsive and phytohormone responsive cis-elements, represented in brown, blue and green, respectively, circle sizes indicate the number of cis-elements.

The upstream promoter regions (1000bp) of the BnGR2R3-MYB genes were predicted for better understanding of the R2R3-MYB genes functions. There were total of 41 responsive cis-acting elements (Figure 6 and Supplementary Table S6), which made up of 22 light responsive elements, 8 phytohormone responses, 6 plant growth development-related responses and 5 abiotic stresses (adversity). Light response elements being the most abundant are present in all genes. Box-4, G-box (light responsiveness), ARE (anaerobic induction) and ABRE (abscisic acid responsiveness) were the most prominent elements, and presence in more than 100 of the 105 BnGR2R3-MYB genes, suggesting that these elements might play an important role in regulating gene expression. A total of 65 (61.9%) genes were found to contain ABRE. Genes containing anaerobic induction, wound response, low temperature response, and drought stress related elements accounted for 55.2%, 26.7%, 24.8% and 17.1%, respectively. In addition, there are 118 elements in all genes involved in the MeJA response. These results indicate the potential function of BnGR2R3-MYB genes in response to various stresses.

Almost all BnGR2R3-MYB genes as obtained in this study contained several cis-acting elements associated with abiotic stresses such as anaerobic induction, drought, low temperature, defense and stress, which is in line with literature (Yamaguchi-Shinozaki and Shinozaki, 2006; Chen et al., 2021). The abiotic stress responsive elements were mostly clustered together, for example, A29 and A30 phylogenetic subgroups in Figure 6 (corresponding to the C32 and C33 subgroups in Figure 5) which were consistent with their functional clustering and might be related to the function of gene groups. This further reflected the accuracy of the results.

Gene ontology (GO) (Ashburner et al., 2000) annotations of 105 BnGMYB proteins is displayed in Supplementary Figure S4 and indicated the proteins involvement in biological process (BP), cellular component (CC) and molecular function (MF). The GO term “binding” (GO: 0005488) best described the greatest number of genes (96, 92.38%), and “single-organism developmental process” (GO: 0044767), “developmental process” (GO:0032502) were significantly enriched in top 20 of biological process. These GO annotations of BnGMYB proteins were in agreement with the experimental findings in other species (Katiyar et al., 2012; Smita et al., 2015; Chen et al., 2017).

### Expression profiling of BnGR2R3-MYB genes with RNA-seq in different tissues

**FIGURE 7:**
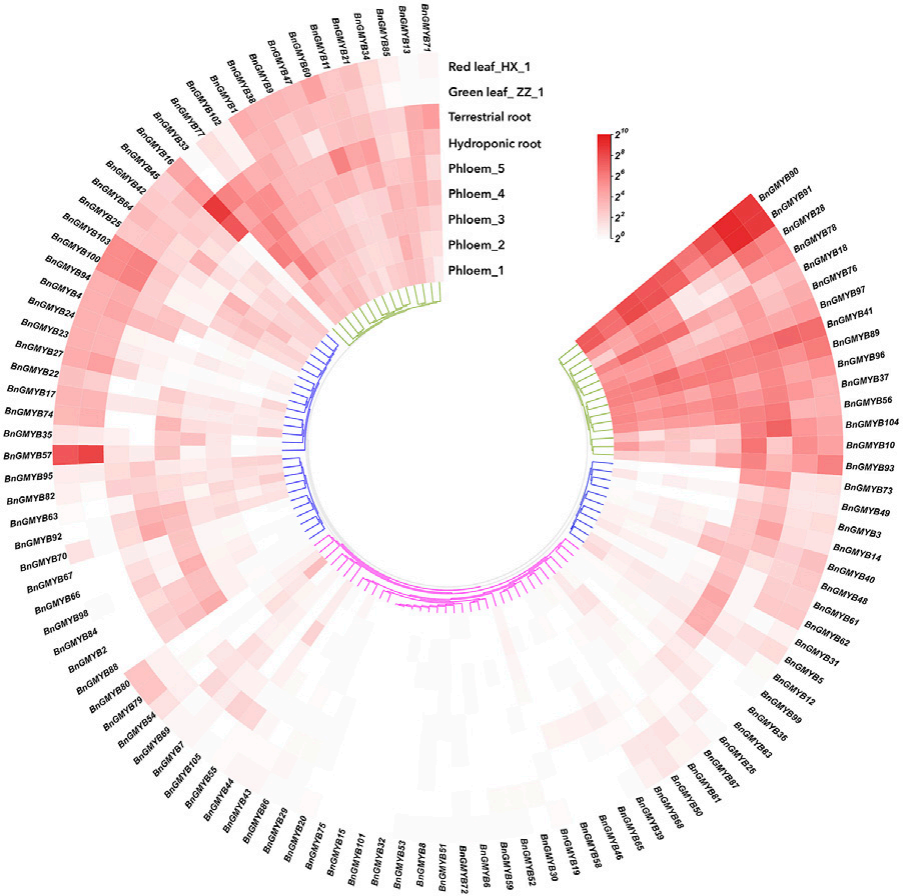
Hierarchical clustering of expression profiles of ramie R2R3-MYB genes in 9 samples including different tissues and developmental stages. Log2(FPKM +1) values were displayed according to the color code. Detailed FPKM values were listed in Supplementary Table S7.

We analyzed transcript levels obtained from transcriptome data of 9 different samples comprising tissues (phloem, root, leaf) and developmental stages of ramie to profile expression of BnGR2R3-MYB. The results (Figure 7 and Supplementary Table S7A) showed most BnGMYBs to have exhibited different expression patterns in line with what was obtained in other plants (Chen et al., 2021). Three BnGMYBs (BnGMYB15; BnGMYB75; BnGMYB101) were not detected in all nine samples, suggesting that they might be pseudogenes, or only expressed in other tissues. Hierarchical cluster analysis of the BnGMYBs from 9 the different samples enabled grouping them into three: high expression, preferential expression, and relatively lower expression. The expression grouping in most cases indicated that the expression patterns of genes were significantly different. However, some closely related MYB genes showed highly similar transcript levels, for example, three members of A30 (*BnGMYB89/90/91*) all belonged to high expression group. A total of 57 genes were expressed in all tissues, and 16 from which showed constitutive expression (FPKM>2). In order to better understand the preferential expression of genes, we filtered genes based on meeting two set rules: FPKM>2 (at least in one tissue); tissues with the highest expression levels should have twice as much expression in at least one of the other tissues. Based on this, 11 genes in phloem_third period, 19 genes in leaf of variety ZZ_1, 22 genes in terrestrial root were found to have exhibited preferential expression over the others (Supplementary Table S7C).

In our previous study, we conducted transcriptomic and non-targeted metabolomic studies of two ramie varieties which have distinct differences in leaf color, HX_1 (red-leaf variety) and ZZ_1 (green-leaf variety), and revealed that the color differences between the duo was due to differences in expression of anthocyanin synthesis pathway related gene, with no clarity in the absolute content of ramie anthocyanin and the function of regulatory gene (e.g., transcription factors) (Feng et al., 2021). In this study, we examined the composition and content of anthocyanin in the two varieties. The results showed that the anthocyanin in ramie was mainly Cyanidin-3-rhamnoside (Supplementary Figure S5). The Cyanidin-3-rhamnoside content in HX_1 was as computed based on the peak value was 3.147 μg/mL, whereas the anthocyanin was almost absent in ZZ_1. Thus, the differences in the leaf color were attributed to this.

**FIGURE 8:**
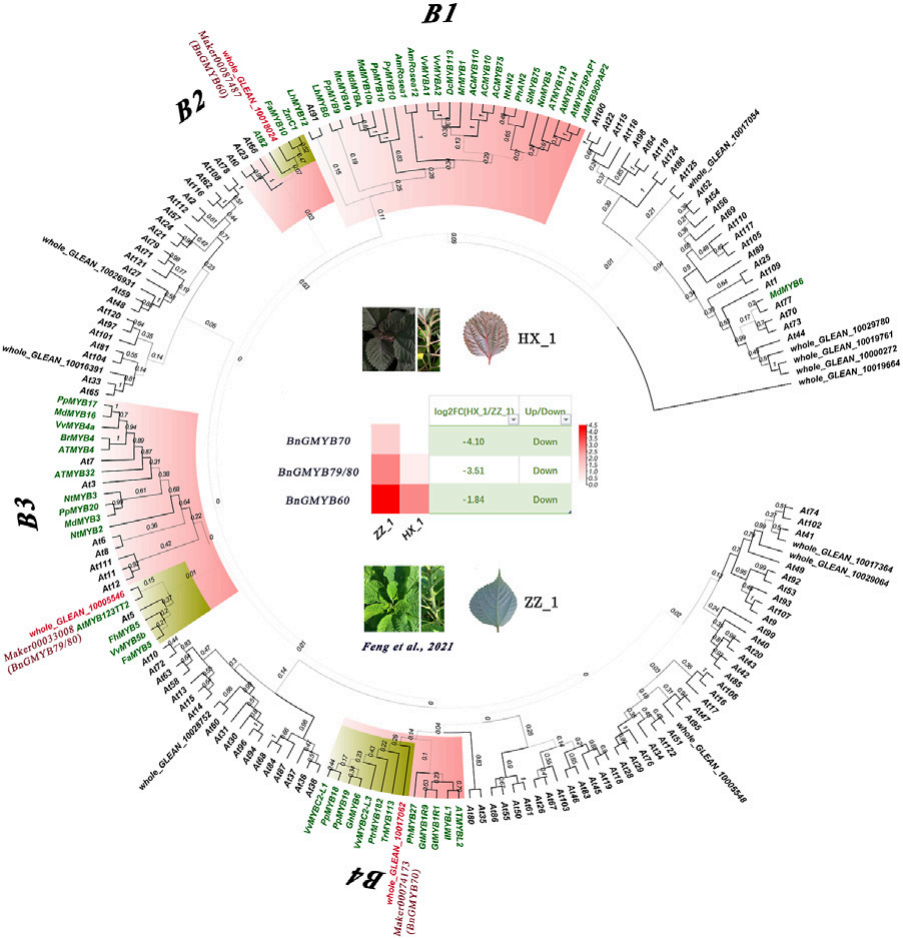
Phylogenetic tree and gene expression analysis of R2R3-MYB transcription factors associated with anthocyanin synthesis.

Several transcription factors including MYB have been reported to interact with key genes involved in anthocyanin synthesis (Lloyd et al., 2017). Fourteen differentially expressed MYB transcription factors searched in the transcriptome data showed 5 to have been upregulated with the remaining 9 downregulated. The highest differential expression ploidy reached up to 17-fold, implying that MYB was very active in two varieties of different colors (Supplementary Table S7B). Therefore, we selected these MYBs for the construction of phylogenetic tree with 50 anthocyanin synthesis-related MYB genes identified in other plants and R2R3-MYBs of the model plant Arabidopsis. As shown in Figure 10, the 50 anthocyanin synthesis related MYBs were grouped into five subgroups, and three of the 14 differentially expressed MYB genes were clustered into these subgroups: *BnGMYB60* (B2), *BnGMYB79/80* (B3), and *BnGMYB70* (B4). All three genes which are homolog of *PtrMYB182* (Yoshida et al., 2015)/*VvMYBC2L3* (Cavallini et al., 2015), *AtMYB123* (*TT2*) (Zimmermann et al., 2004), and *AtMYB82* (Zhou et al., 2020) involve anthocyanin synthesis repression were downregulated in HX_1 (Figure 8). No ramie MYB was found in the B1 subfamily that positively regulates anthocyanin, and therefore anthocyanin synthesis in ramie is not regulated by the B1 subgroup MYB transcription factors. It will be envisaged that, upregulation of *BnGMYB60* (B2), *BnGMYB79/80* (B3), and *BnGMYB70* (B4) caused the inhibition of ZZ_1 anthocyanin synthesis, which ultimately affected color formation.

### Expression profiles of BnGMYBs under Cd^+2^ stress

Ramie is a promising plant for remediation of heavy metal contaminated land and has high cadmium tolerance and accumulation capacity (She et al., 2015). An increasing number of studies have linked R2R3-MYB genes to various stress responses and regulation. However, there was not much information on the involvement of MYB genes in cadmium tolerance response in ramie. We thus screened 11 MYBs candidates by combining RNA-seq expression profiles, homologous gene relationships, as well as their expression patterns under cadmium stress.

**FIGURE 9:**
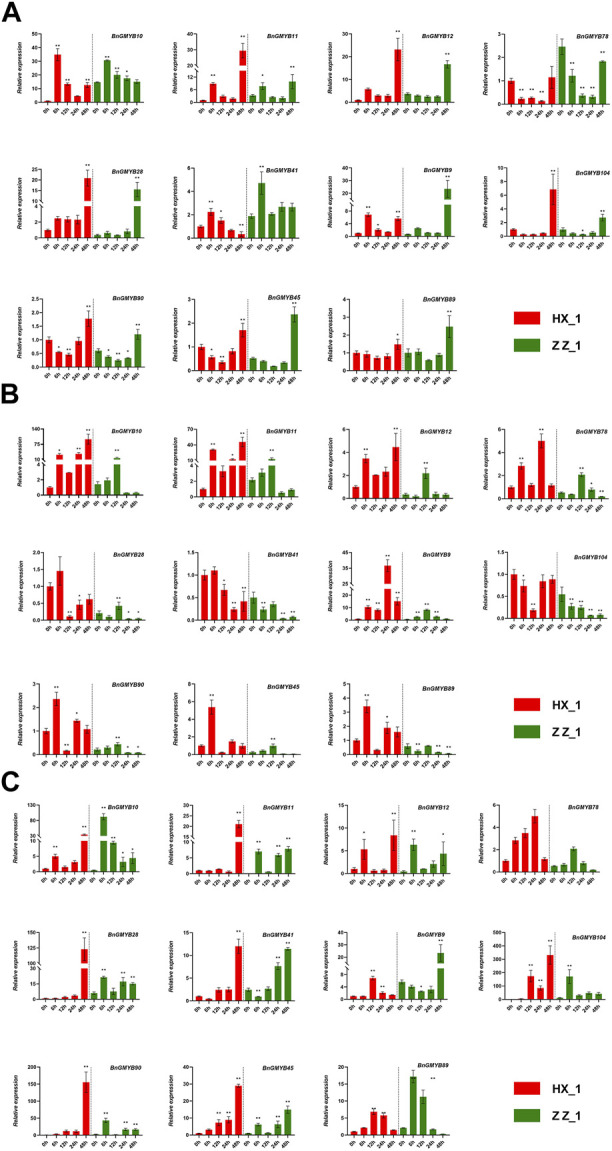
The relative expression levels of selected MYB genes in two ramie varieties in different tissue sites (root, stem, leaf) at different periods under Cd^+2^ treatment (0, 6, 12, 24, 48 h). **(A)** represents root; **(B)** represents stem; **(C)** represents leaf. The error bar represents the standard deviation of the three biological duplicates. * represents significant difference (*p* < 0.05), ** represents extremely significant difference (*p* < 0.01).

We analyzed them in three dimensions: treatment time, species, and tissue site. The results (Figure 9) showed varied expression patterns of the BnGMYB genes under different Cd^2+^ treatment times with four different trends of continuously rising, rise and fall, continuously falling, and rising after falling. Majority of the genes showed characteristics fall upon treatment before rising. The expression tendency of 11 genes were consistent in the two ramie cultivars, while some genes responded more strongly in HX_1 than in ZZ_1, such as *BnGMYB28*, *BnGMYB9*, *BnGMYB78*, indicating that the response mechanisms to Cd^2+^ stress is similar in the two cultivars. The degree of response in different parts of the plant under the same stress was also different, however, all the genes were upregulated in the leaves on cadmium treatment. Combining the results of different treatment times and tissue sites, we found that genes such as *BnGMYB9/11/41/89* showed a bottom-up response pattern in the order (root-stem-leaf) which may relate to cadmium transport and stress signaling.

**FIGURE 10:**
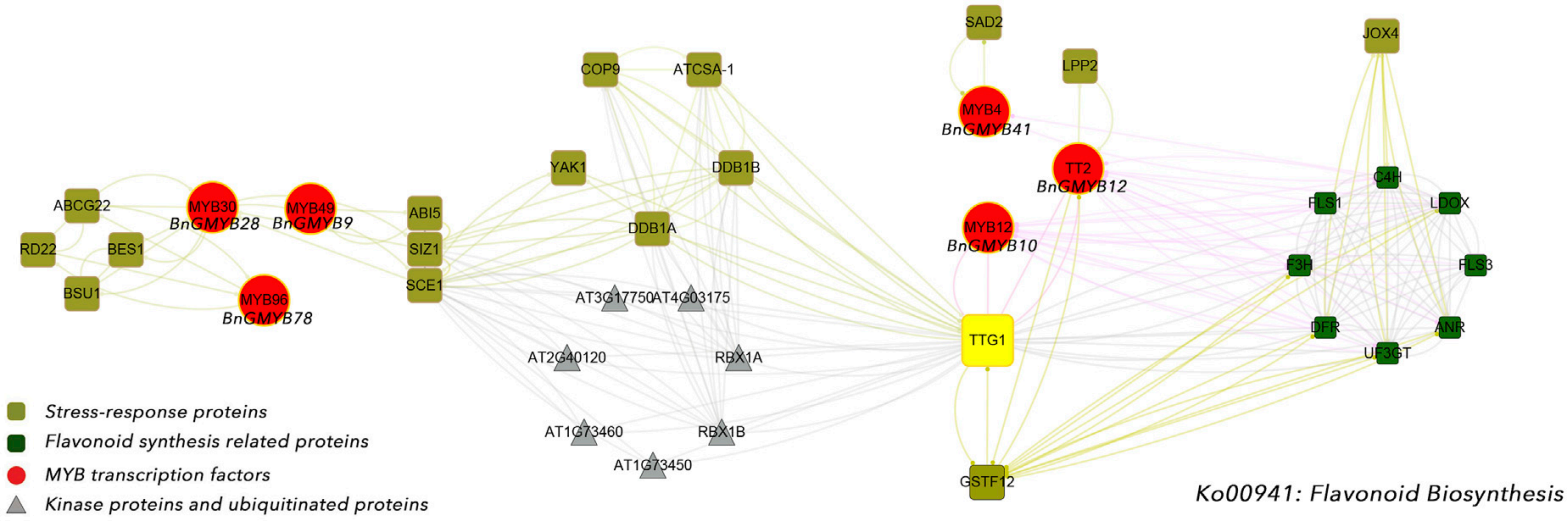
The protein-protein interaction network for BnGR2R3-MYB proteins based on their Arabidopsis orthologs. The red solid circles indicated cadmium stress-related MYB transcription factors, the olive squares represented stress-response proteins, and the green squares were flavonoid synthesis related proteins.

The expression of *BnGMYB10/12/41* in roots, stems and leaves all increased more than 10-fold after cadmium stress, and phylogenetic analysis placed these three genes into the flavonoid synthesis MYB subgroup (C1), suggesting a potential association between flavonoid synthesis and cadmium tolerance (Dubos et al., 2010). For further understanding the association of flavonoid synthesis and cadmium stress, we selected *AtMYB4* (Agarwal et al., 2020), *AtMYB30* (Fichman et al., 2020), *AtMYB49* (Zhang et al., 2019), and *AtMYB96* (Seo et al., 2011) which are respectively homologs of *BnGMYB41, BnGMYB28, BnGMYB9, BnGMYB78* with established role in defense responses, and *AtMYB4* (Agarwal et al., 2020), *AtMYB12* (Mehrtens et al., 2005), *TT2* (Baudry et al., 2004) (homologs of *BnGMYB10*/*BnGMYB12*/*BnGMYB41*, respectively) involved in regulating flavonoid synthesis pathway in *Arabidopsis thaliana* for protein interaction network analysis (Figure 10). These MYB TFs were found to interact with flavonoid biosynthetic genes, in addition to being tightly linked to some resistance proteins. *BnGMYB12* and *BnGMYB41* interacted with *LPP2* (lipid phosphatase 2) (Pierrugues et al., 2001), *SAD2* (Zhao et al., 2007) (sensitive protein to ABA and drought 2), respectively. A number of proteins in the flavonoid metabolic pathway have been reported to interact with jasmonic acid-induced oxygenase 4 (*JOX4*) (Smirnova et al., 2017). We also found that *BnGMYB9/28/78* interacts with many important stress resistance proteins such as *SIZ1* (Miura et al., 2020), *RD22* (Ma et al., 2018), *YAK1* (Kim et al., 2016), *ABI5* (Ma et al., 2018), *etc.* Interestingly, we found an interface between the flavonoid metabolic pathway and cadmium stress, the *TGG1* (thioglucoside glucohydrolase 1) node connecting two possible stress coping mechanisms. This finding provides a basis for understanding the association of flavonoid synthesis and stress regulation in ramie.

## Conclusion

A total of 105 BnGR2R3-MYBs unevenly distributed among 14 chromosomes, phylogenetically divided into 35 distinct subfamilies, were identified in ramie. The segmental duplication events especially prominent in distal telomeric regions, played a crucial role in the expansion of BnGR2R3-MYB gene family. The result showed highest orthologous gene pairs between ramie and *Apocynum venetum* (58) than other plants including Arabidopsis. Expression analysis led to the identification of tissue preferential and cadmium stress responsive expression patterns of the BnGR2R3-MYB genes. Additionally, putative functions of ramie MYB genes were assigned based on the phylogenomic results and gene expression data. We performed comparative transcriptome analysis between HX_1 and ZZ_1 coupled with phylogenetic and UPLC-QTOF-MS analysis and identified *BnGMYB60*, *BnGMYB79/80* and *BnGMYB70* as potential anthocyanin biosynthesis repressor genes. Expression profiling and analysis of some selected BnGMYB revealed *BnGMYB9/10/12/28/41/78* as potential cadmium stress regulators and *BnGMYB10/12/41* in addition, as regulatory genes of flavonoid biosynthesis which is in accordance their Arabidopsis annotated homologues. Thus, this study provided significant information into MYB regulatory genes in ramie and may serve as a foundation for genetic enhancement and increased productivity.

## Materials and methods

### Identification of MYB protein in Boehmeria nivea (L.) gaudich

For the comprehensive identification of the ramie MYB TFs, 168 MYB family sequences and 97 MYB-related family sequences (*A. thaliana*), 208 MYB family sequences and 85 MYB-related family sequences (*G. arboreum*), 130 MYB family sequences and 106 MYB-related family sequences (*Oryza sativa*) were retrieved from the PlantTFDB (http://planttfdb.gao-lab.org/). The protein sequences of these species were then used as reference to filter the possible sequence of target specie by Blast Wrapper, with expectation cut-off value (E-value) of e−5 set as threshold significance. The sequences obtained were aligned to Swissport Database (https://www.expasy.org/resources/uniprotkb-swiss-prot)/Reference Species Whole Protein Sequence Library (https://www.ncbi.nlm.nih.gov/refseq/) and screened by annotation information. The resulting datasets of MYB sequences were confirmed based on the completeness of MYB domains using Pfam, CDD and SMART with an E-value set at 0.01. To correct for deletion of some conserved sites, FGENESH-M (http://www.softberry.com/berry.phtml) was used to predict multiple variants potential genes in genomic DNA. All the resulting ramie R2R3-MYB proteins were manually inspected to ensure that it contained two complete MYB domains and ultimately identified and classified as members of the MYB family in *Boehmeria nivea*. Length of sequences, molecular weights, GRAVY and pI of the MYB proteins were obtained using the ExPASy online tool (Wilkins et al., 1999)and subcellular localization predicted using the pLoc-mPlant tools for Batch Prediction.

### Sequence analysis and structural characterization of BnGR2R3-MYB genes

Multiple sequence alignments of the MYB domains sequences were performed using MEGA-X with default parameters. The deduced amino acid sequences of the MYB motifs were adjusted manually in Jalview software (Waterhouse et al., 2009) and WEBLOGO (Crooks et al., 2004) used to show up the sequence logos of R2 and R3 MYB domain repeats. The exon-intron organizations of the BnGR2R3-MYB genes, including intron distribution patterns, phases, intro-exon boundaries and highlighted region of the MYB domains were graphically displayed with the aid of TBtools (Chen et al., 2020). The Conserved motifs of the ramie MYB proteins were predicted by using the MEME (Bailey et al., 2009) version 5.1.0 with optimized parameters: zero or one per sequence; maximum number of motifs set at 20, and visualized in TBtools.

### Analyses of chromosome distribution, gene duplication and synteny for BnGR2R3-MYB genes

The detailed chromosome distribution information of each BnGR2R3-MYB genes was obtained from the ramie genome annotation documents. Circos (Krzywinski et al., 2009) was used to locate all the BnGR2R3-MYB genes on the ramie chromosome. Tandem, segmental duplication and collinearity within species were obtained by using Multiple Collinearity Scantoolkit (MCscanX) (Wang et al., 2012). Synteny blocks of the orthologous R2R3-MYB genes between ramie and other species were obtained also using MCscanX and both results visualized in TBtools (Chen et al., 2020). Non-synonymous and synonymous substitution of each duplicated BnGMYB genes were calculated using KaKs_Calculator 2.0 (Wang et al., 2010).

### Phylogenetic analysis and classification of ramie BnGR2R3-MYB proteins and WGDI analysis

Multiple sequence alignments of the R2R3-MYB proteins from ramie and *Arabidopsis thaliana* were performed using MEGA 7.0 with default parameters (Kumar et al., 2016) and neighborhood linkage (Guo et al., 2019) phylogenetic tree constructed with the following parameters: Poisson model; pairwise deletion; and 1,000 bootstrap replications. R2R3-MYB proteins from other species [Arabidopsis (Holub, 2001), rice (Katiyar et al., 2012), maize (Du et al., 2012b), tomato (Li et al., 2016), pineapple (Liu et al., 2017)] were obtained based on the description in corresponding literatures, and adopted same.

To establish WGD events, paralogous genes of ramie were detected using all-vs-all homology searches in BLASTP with an E-value threshold of 1e-5. Syntenic blocks within a genome were identified based on the detected homologous gene pairs using MCscan.

### Identification of the cis-elements of BnGR2R3-MYB genes and GO annotation

The promoter regions of the BnGR2R3-MYB genes, 1,000 bp genomic DNA upstream sequences of each of the 105 BnGR2R3-MYB were selected, and the cis-elements predicted using PlantCare (http://bioinformatics.psb.ugent.be/web tools/plantcare/html/). Response class elements such as light-responsive, plant growth, stress-responsive and phytohormone-responsive were filtered and the cis-acting elements visualized in TBtools, and displayed with a heatmap. Following blasting in BLASTP against Swissport database, functional annotation of BnGMYB proteins was performed in Blast2GO Tool and subsequent mapping Gene Ontology (GO) terms, and visualization with R ggpolt2.

### Expression patterns of BnGR2R3-MYB genes in the representative tissues of the ramie

Transcriptome data of 9 samples comprising phloem, root and leaf tissues as well as, developmental periods and growth condition (hydroponics or terrestrial) obtained from Genome Sequence Archive database were used. Transcript abundance of the BnGR2R3-MYB genes was calculated as fragments per kilobase of exon model per million mapped reads (FPKM). Log2(FPKM +1) values were displayed according to the color code. Detailed FPKM values were listed in Supplementary Table S7.

### Determination of anthocyanin content by UPLC-QTOF-MS

Anthocyanin content was determined following the method of Wang et al. (2020). Leaves samples of HX_1 and ZZ_1 (0.4 g each) was separately extracted at 4°C for 12 h using methanol/formic acid (4 mL) mixture (9:1, v/v). The extracts were then centrifuged for 20 min at 3,900 rpm and the supernatant filtered using a 0.22 μm nylon membrane into brown glass tube. The UPLC-QTOF-MS analysis was performed on Waters ACQUITY UPLC I-Class-Xevo G2-XS QTOF/PDA eLambda Detector equipped with an ACQUITY UPLC CSH C18 (2.1 mm × 100 mm i.d., 1.7 μm) column. Acetonitrile with 5% formic acid and water with 5% formic acid were respectively used as mobile phase A and B at 190 nm–800 nm wavelength photo-diode array (PDA). It was operated at positive ion mode for mass and the injection volume set at 1 μL for all samples. ESI source parameters were according to literature (Wang et al., 2020) and MS/MS for fragments ranged from 50 m/z to 1,000 m/z in a continuum mode. Ramp was in the range of 6 V–80 V. Peonidin-3-glucoside chloride (15 μg) was used as reference standard.

### Stress treatment (Cd^2+^) of ramie under hydroponic conditions

Two ramie varieties (ZZ_1; HX_1) were used for cadmium stress treatment. The two species have conspicuous leaf color attributed by different anthocyanin content (Feng et al., 2021) Shoots from each of the varieties at similar growth stage during the same period were selected for hydroponic cutting according to Gao et al. (2018). These were monitored for 15 days and seedlings with inconsistent growth were removed. CdCl_2_ to a final concentration of 50 mg/L was added and three separate biological replicates were sampled at 0, 12, 24, and 48 h after the treatments. All samples were frozen quickly in liquid nitrogen and stored at −80°C until used.

### BnGMYB protein–protein interaction network

Orthologous relationships of eight selected cadmium responsive MYB regulatory genes were determined between *A. thaliana* and ramie using OrthoVeen2 (Xu et al., 2019). Prediction of interactions between BnGMYB proteins and other proteins based on the Arabidopsis homologs was obtained using the online program STRING version 11.5 with high confidence>0.700 (Szklarczyk et al., 2017), filtered genes were used to construct the correlation network. The interaction network was visualized in Cytoscape v3.8.2.

### RNA isolation and expression analysis of BnR2R3-GMYB genes

Total RNA was extracted using EasySpin Plus plant RNA rapid extraction Kit (Aid-lab Biotechnologies Co., Ltd.). The RNA was reverse-transcribed (Thermo Scientific RevertAid First cDNA Synthesis Kit) into cDNA and quantitative RT-PCR (qPCR) analysis conducted using gene specific primers (Supplementary Table S8]). 18 s gene was used as internal control. The qPCR was conducted using SYBR Green Premix Pro Taq HS qPCR Kit (Accurate Biotechnology Co., Ltd.) on a CFX96 Touch Deep Well Real-Time PCR System (Bio-Rad) according to standard procedure. Relative transcript levels were calculated using the 2^−ΔΔCt^ formula and the result displayed using histograms drawn with GraphPad Prism 8 software and all the histograms merged using Adobe Photoshop (2020) software. All qPCR analyses were performed with three biological and technical replications.

## Data Availability

The original contributions presented in the study are included in the article/Supplementary Material, further inquiries can be directed to the corresponding author/s.

## Supplementary material

SUPPLEMENTARY FIGURE S1 Phylogenetic relationships (neighbor-joining), gene structure and architecture of conserved protein motifs in BnGR2R3-MYB genes.

SUPPLEMENTARY FIGURE S2 Phylogenetic analysis of the R2R3-MYB family in six different plant species.

SUPPLEMENTARY FIGURE S3 Comparative genomic analysis—WDGI. The point with the best homology is red (The highest similarity). The diagonals are not self-alignments of genes, but tandem repeats.

## Abbreviations

HTH, helix–turn–helix; MEME, Multiple Em for Motif Elicitation; NJ, Neighbor–Joining; ORF, open reading frame; GSDS, Gene structure display server; PlantTFDB, Plant Transcription Factor Database; RNA-seq, RNA-sequencing; SMART, Simple Modular Architecture Research Tool; TAIR, The Arabidopsis Information Resource; TFs, Transcription Factors; qPCR, Quantification real-time PCR; MWs, Molecular weights; MEME, Multiple Expectation maximization for Motif Elicitation; pIs, Isoelectric points; GRAVY, Grand average of hydropathicit; HX_1, Hongxuan No. 1 variety; ZZ_1, Zhongzhu No. 1 variety.
