# Supplementary material for: Genome-wide analysis of R2R3-MYB transcription factors in Boehmeria nivea (L.) gaudich revealed potential cadmium tolerance and anthocyanin biosynthesis genes
Source: Front Genet. 2023 Feb 21;14:1080909. doi: 10.3389/fgene.2023.1080909 (PMC9989182; doi:10.3389/fgene.2023.1080909)

Figure S1: Phylogenetic relationships (neighbor-joining), gene structure and architecture of conserved protein motifs in BnGR2R3-MYB genes

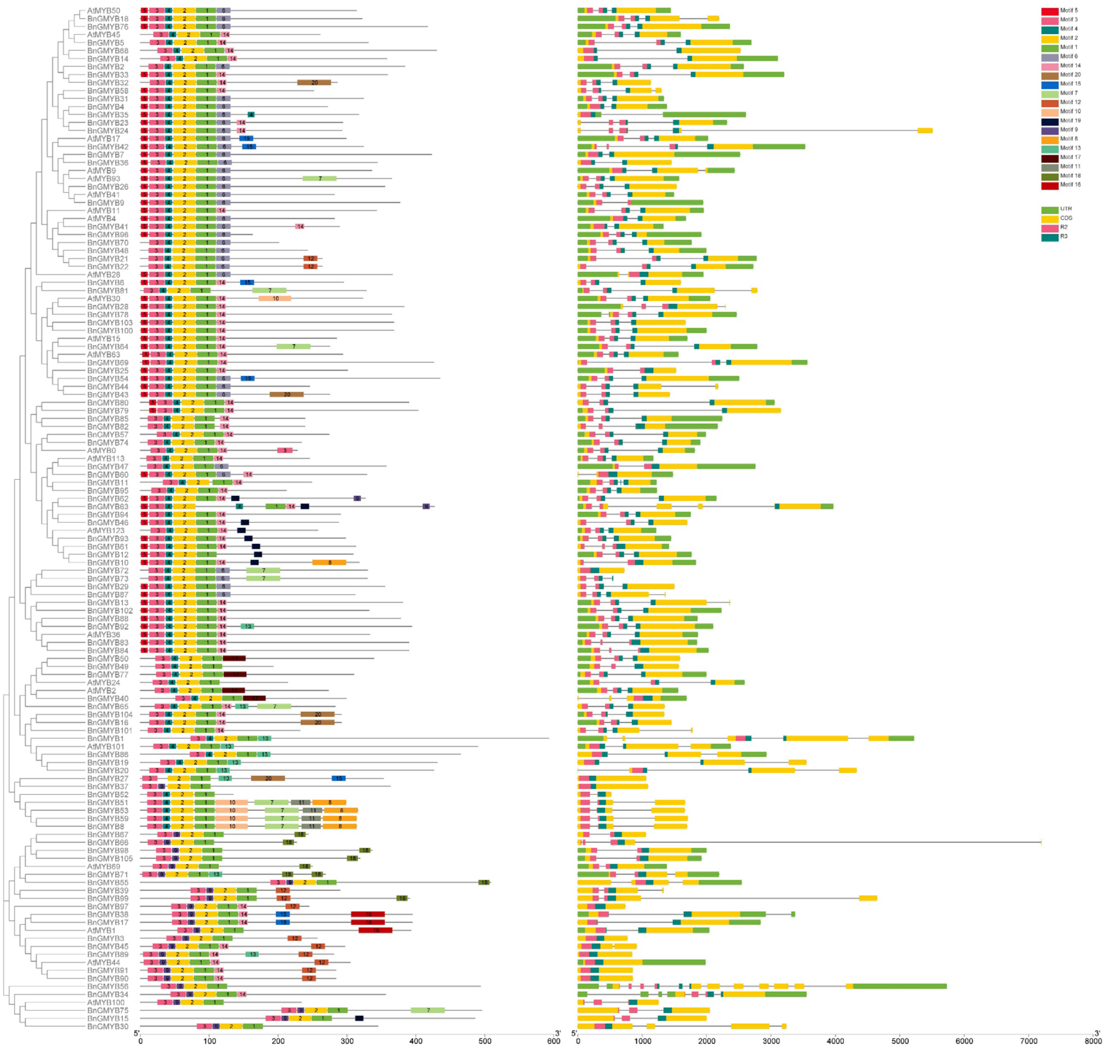

Supplement: Supplementary file 4 [file Image1.pdf]
