# Supplementary material for: Genome-wide analysis of R2R3-MYB transcription factors in Boehmeria nivea (L.) gaudich revealed potential cadmium tolerance and anthocyanin biosynthesis genes
Source: Front Genet. 2023 Feb 21;14:1080909. doi: 10.3389/fgene.2023.1080909 (PMC9989182; doi:10.3389/fgene.2023.1080909)
Supplement: Supplementary file 6 [file Image3.pdf]

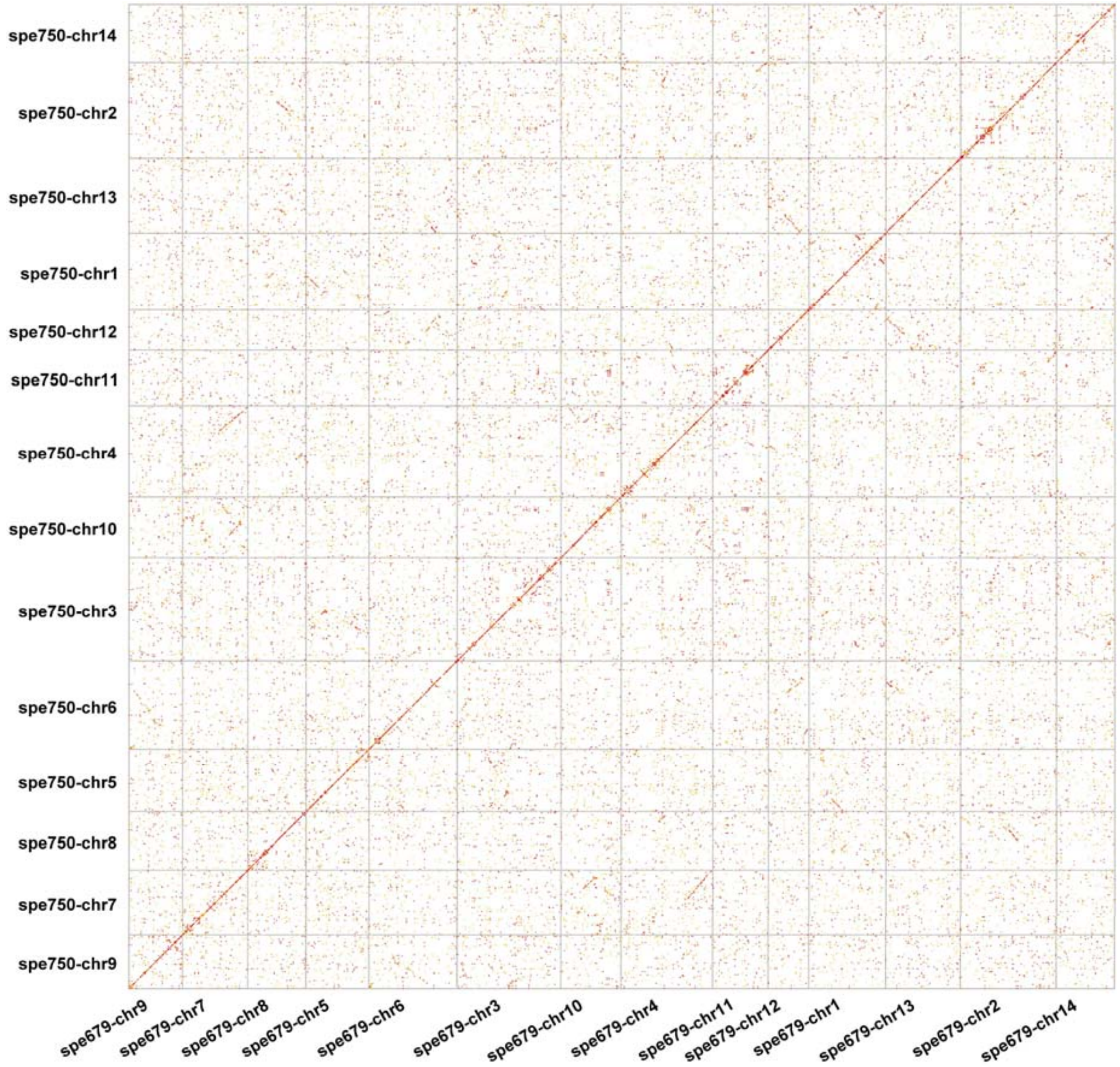

Figure S3: Comparative genomic analysis—WDGI.

The point with the best homology is red (The highest similarity). The diagonals are not self-alignments of genes, but tandem repeats.
